# Diurnal Variations in Blood Flow at Optic Nerve Head and Choroid in Healthy Eyes: Diurnal Variations in Blood Flow

**DOI:** 10.1097/MD.0000000000000519

**Published:** 2015-02-13

**Authors:** Takeshi Iwase, Kentaro Yamamoto, Eimei Ra, Kenta Murotani, Shigeyuki Matsui, Hiroko Terasaki

**Affiliations:** From the Department of Ophthalmology (TI, KY, ER, HT), Nagoya University Graduate School of Medicine; Center for Advanced Medicine and Clinical Research (KM), Nagoya University Hospital; and Department of Biostatistics (SM), Nagoya University Graduate School of Medicine, Nagoya, Showa-ku, Japan.

## Abstract

To investigate the diurnal variations of the ocular blood flow in healthy eyes using laser speckle flowgraphy (LSFG), and to determine the relationship of the diurnal variations between the ocular blood flow and other ocular parameters.

This prospective cross-sectional study was conducted at Nagoya University Hospital. We studied 13 healthy volunteers whose mean age was 33.5 ± 7.6 years. The mean blur rate (MBR), expressing the relative blood flow, on the optic nerve head (ONH) and choroidal blood flow was determined by LSFG (LSFG-NAVI) every 3 hours from 6:00 to 24:00 hours. The intraocular pressure (IOP), choroidal thickness measured by enhanced depth imaging optical coherence tomography, systolic (SBP) and diastolic (DBP) blood pressure, and heart rate (HR) in the brachial artery were also recorded. We evaluated the diurnal variations of the parameters and compared the MBR to the other parameters using a linear mixed model.

The diurnal variations of the MBR on the ONH varied significantly with a trough at 9:00 hours and a peak at 24:00 hours (*P* < 0.001, linear mixed model). The MBR of choroid also had significant diurnal variations with a trough at 15:00 hours and a peak at 18:00 hours (*P* = 0.001). The IOP (*P* < 0.001), choroidal thickness (*P* < 0.001), SBP (*P* = 0.005), DBP (*P* = 0.001), and HR (*P* < 0.001) also had significant diurnal variations. Although the diurnal variation of the MBR on the ONH was different from the other parameters, that on the choroid was significantly and positively correlated with the DBP (*P* = 0.002), mean arterial pressure (*P* = 0.023), and mean ocular perfusion pressure (*P* = 0.047).

We found significant diurnal variations in the ONH and choroidal blood flow. Although the ONH blood flow had its own diurnal variation because of strong autoregulation, the choroidal blood flow was more likely affected by systemic circulatory factors because of poor autoregulation.

## INTRODUCTION

Knowledge about ocular blood flow is important for understanding pathological conditions and the treatment of various ocular diseases. The blood flow on the optic nerve head (ONH) has been reported to be reduced in some ocular diseases, for example, glaucoma,^1–3^ and impaired choroidal blood flow is associated with various ocular diseases, for example, choroidal neovascularization,^4^ retinitis pigmentosa,^5,6^ and glaucoma.^7,8^ It has been reported that blood flow on the ONH is autoregulated for fluctuations of the IOP and systemic blood pressure.^9^ In contrast, the choroidal vessels have been reported to have poor autoregulation, and changes in the ocular perfusion pressure directly affect the choroidal blood flow.^10^

It is well established that diurnal variations are present in various anatomic and physiological parameters of the eye, for example, the intraocular pressure (IOP),^11–14^ corneal thickness,^15–17^ corneal topography,^15,18^ pupillary diameter, anterior chamber biometrics, and choroidal thickness.^14,19,20^ Even though investigations of the diurnal changes of the ONH and choroidal blood flow are important for evaluating blood flow of ocular diseases accurately, there are few reports describing any variations in the intraocular blood flow.^9,21,22^

A variety of techniques for measuring retinal blood flow have been developed, including fluorescein angiography,^23^ the radioactive microsphere technique,^24^ hydrogen clearance method,^25^ and laser Doppler velocimetry.^26,27^ However, all of them have various limitations in evaluating diurnal variation of ocular blood flow because it is difficult for those to follow the blood flow of the same sites at different times.

Laser speckle flowgraphy (LSFG) is a method that can measure the relative blood flow of the vessels on the ONH and choroid noninvasively without the use of contrast agents.^28^ In addition, the LSFG-NAVI instrument (Softcare, Fukuoka, Japan) can measure the same area of the fundus repeatedly because the instrument automatically stores the topographic features of the retinal vasculature. The reproducibility of measurements of blood flow in ocular tissues by the LSFG method is high, and the measurement time is shorter than that of laser Doppler flowmetry.^29,30^ The LSFG-NAVI is therefore suitable for monitoring changes in ocular tissue circulation at the same site in the same eye at various times during the day or the disease process.^31^

The purpose of this study was to determine the diurnal variations in the ONH and choroidal blood flow simultaneously in healthy eyes, and to investigate the correlation of the diurnal variations between the blood flow and systemic and ocular factors.

## METHODS

### Subjects

In this prospective cross-sectional study, the procedures used were approved by the Ethics Committee of Nagoya University Hospital and were conducted at Nagoya University Hospital. The procedures conformed to the tenets of the Declare of Helsinki. An informed consent was obtained from the subjects after an explanation of the nature and possible consequences of the measurements. Twenty-six eyes of 13 Japanese healthy men volunteers with no ophthalmic or systemic diseases were studied. All subjects had a best-corrected visual acuity (BCVA) of ≥20/20 and were examined to determine if any ocular disease was present. Slit-lamp examinations and indirect ophthalmoscopy were used to examine the anterior and the posterior segments of the eye. Subjects were also screened for any medical condition that could influence the hemodynamic of the eye such as diabetes, hypertension, arrhythmia, and vascular diseases.

The exclusion criteria included the presence of any macular abnormalities such as choroidal neovascularization, asymptomatic pigment epithelial detachment, or whitish myopic atrophy, smoking, and history of intraocular surgery.

The relative blood flow was determined by LSFG-NAVI. The subfoveal choroidal thickness, IOP, systolic blood pressure (SBP), diastolic blood pressure (DBP), and heart rate (HR) were also measured every 3  hours from 6:00 to 24:00 hours (Table 1). Because the intake of alcohol^32^ and caffeine^33^ can influence the IOP, all participants were asked to abstain from alcoholic and caffeinated beverages from the evening before and for the day of the study. Additionally, all participants were asked to abstain from consuming any food 2 hours before each experiment. All of the examinations were performed in the sitting position. Each subject rested for 10 to 15 minutes in a quiet room before the tests, and each experimental session was completed within 15 minutes.

**TABLE 1:**
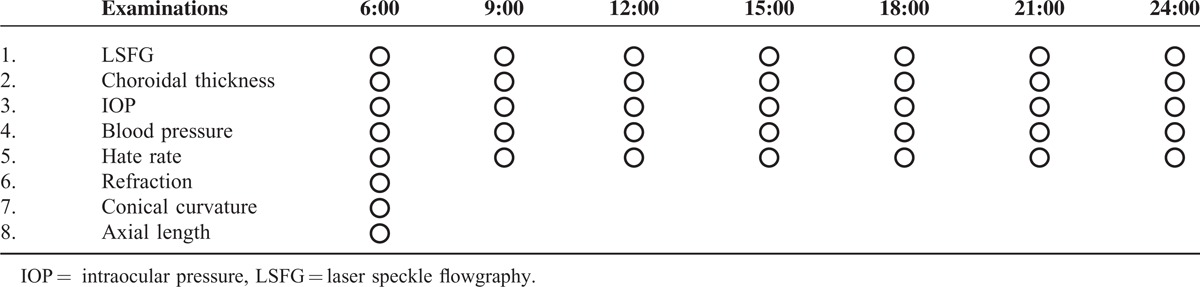
Examination Schedule

### Demographic Data

The demographic data of all of the subjects are shown in Table 2. Thirteen men with a mean age of 33.5 ± 7.6 years and range of 28 to 47 years were studied. The mean refractive error (spherical equivalent) was −4.2 ± 1.8 diopters (D) with a range of −0.5 to −6.25 D in the right eye and −4.4 ± 1.8 D with a range of −1.0 to −6.5 D in the left eye. The mean axial length was 25.81 ± 0.78 mm with a range of 24.59 to 27.38 mm in the right eye and 25.96 ± 0.94 mm with a range of 24.51 to 27.54 mm in the left eye. The values of the IOP, subfoveal choroidal thickness, SBP, DBP, and HR measured at 6:00 hours were taken to be the baseline values (Table 3).

**TABLE 2:**
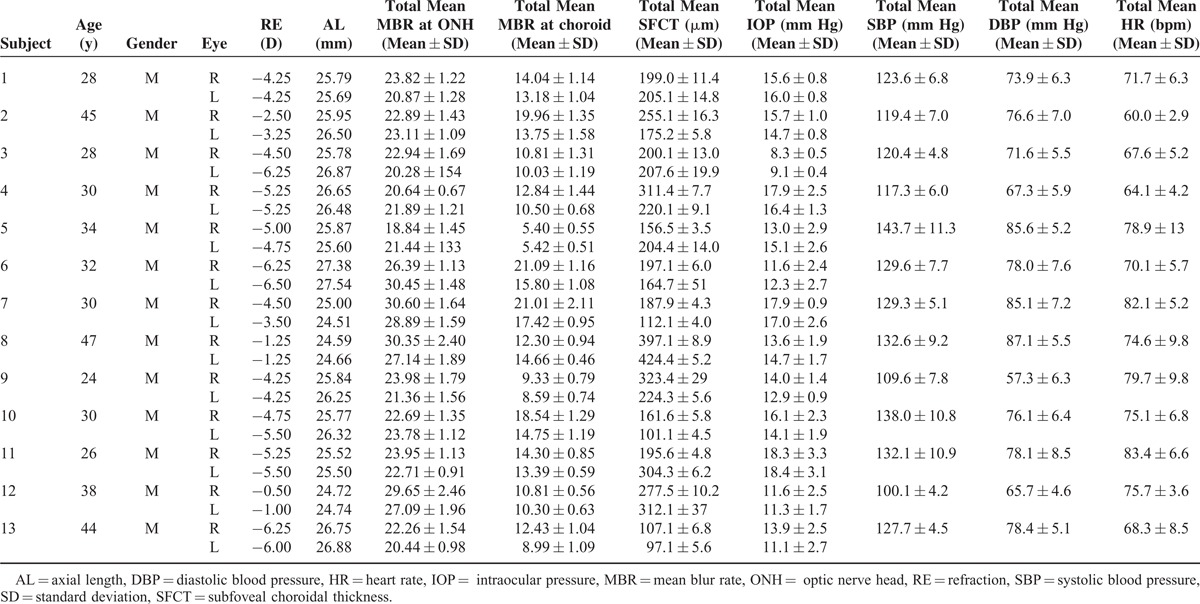
Subject Demographic, Ocular Biometric Parameter, and Systemic Factors

**TABLE 3:**
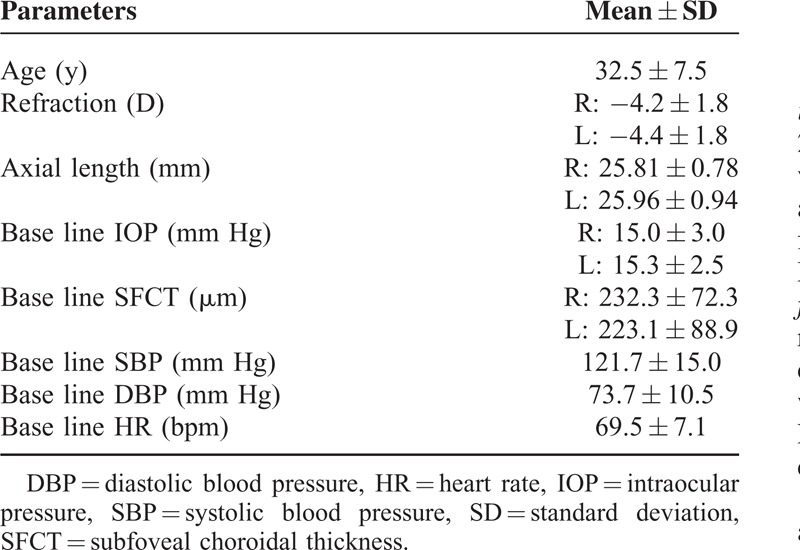
Demographic Characteristics of Subjects Undergoing Examinations

### Laser Speckle Flowgraphy

The LSFG-NAVI was used to determine the relative ocular blood flow. The principles of LSFG have been described in detail.^34–38^ Briefly, this instrument consists of a fundus camera equipped with an 830 nm diode laser and a charge-coupled camera (750 width × 360 height pixels). After switching on the laser, a speckle pattern appears due to the interference of the light scattered from the illuminated tissue. The mean blur rate (MBR) is a measure of the relative blood flow, and it is determined by examining the pattern of the speckle contrast produced by the interference of the laser light that is scattered by the movement of the blood cells in the ocular blood vessels.^39^ The MBR images are acquired at a rate of 30 frames/s over a 4-second period. The embedded analysis software then synchronizes all of the MBR images with each cardiac cycle, and the averaged MBR of a heartbeat is displayed as a heartbeat map.

**FIGURE 1:**
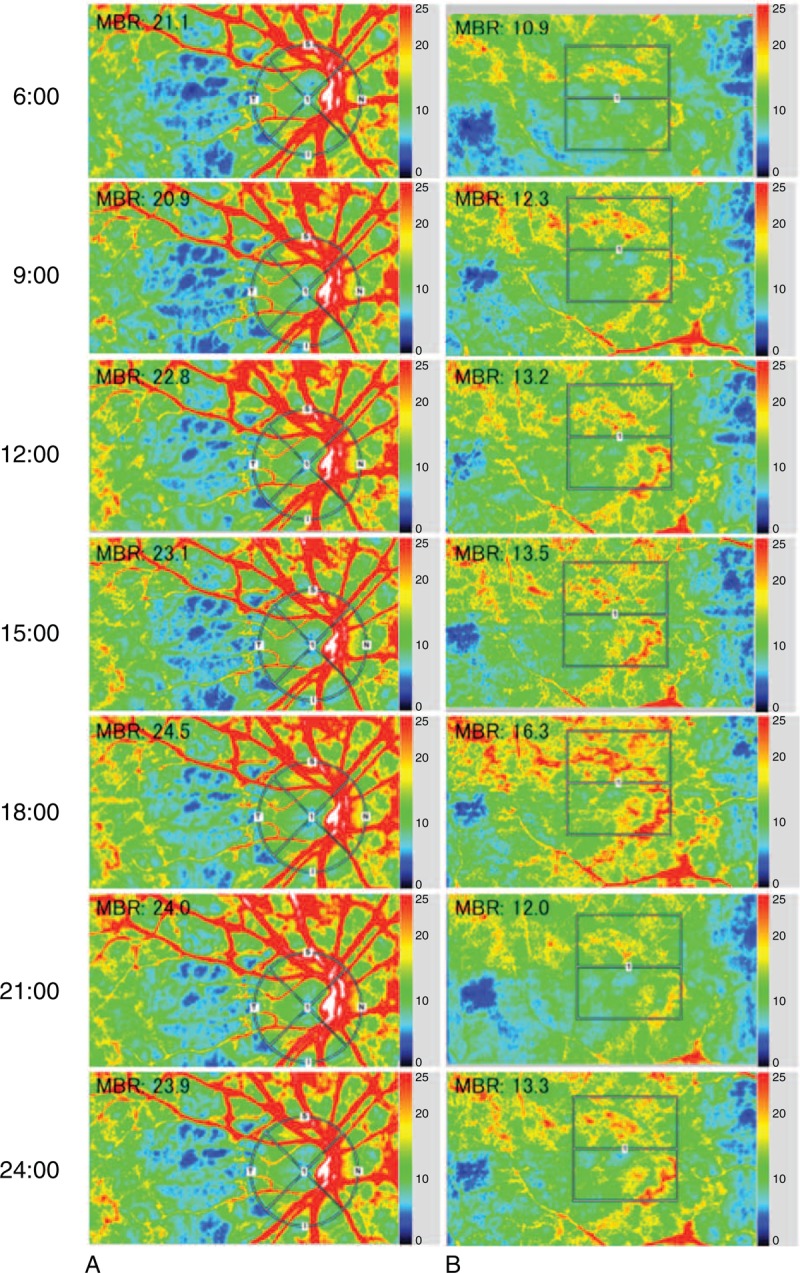
Composite color maps using the mean blur rate (MBR) as measured by laser speckle flowgraphy (LSFG). The red color indicates high MBR and the blue color indicates low MBR. To measure MBR on the optic nerve head (ONH) blood flow and choroidal blood flow, a circle was set around the ONH (A) and a rectangle was set at the macula (B). The MBR was measured every 3 hours from 6:00 to 24:00 hours to evaluate the diurnal fluctuations.

To evaluate the changes in the ONH and choroidal blood flow, a circle was set surrounding the ONH (Figure 1A) and a rectangle (250 × 250 pixels) was placed around the macula (Figure 1B). The “vessel extraction” function of the software then identified the vessel and tissue areas on the ONH, so that the MBR could be assessed separately. We evaluated the MBR in the 3 parts, namely, overall, vessels, and tissue areas on the ONH. The software in the instrument was able to track the eye movements during the measurement period.

The LSFG was measured 2 times at each time point in all of the eyes. The average of MBR values was calculated for each circle or rectangle using the LSFG Analyzer software (V.3.0.47).

### Optical Coherence Tomography (OCT)

Choroidal images were obtained by spectral-domain OCT (SD-OCT; Spectralis OCT, Heidelberg Engineering, Heidelberg, Germany). The SD-OCT instrument was placed close enough to the eye to obtain inverted images as described in detail.^40^ The choroidal thickness at the fovea was measured as the distance from the hyperreflective retinal pigment epithelium line to the choroid–sclera border with the caliper tool on the SD-OCT instrument. Two clinicians who were masked to the other findings measured the thicknesses.

### Other Examinations

The refractive error (spherical equivalent) was measured with an autorefractometer (KR8900; Topcon, Tokyo, Japan), and the axial length was measured by partial optical coherence inferometry (IOLMaster; Carl Zeiss Meditec, La Jolla, CA). The IOP was measured with a handheld tonometer (Icare; Tiolat Oy, Helsinki, Finland). The SBP, DBP, and HR were measured by an automatic sphygmomanometer (CH-483C; Citizen, Tokyo, Japan).

### Hemodynamics

An earlier study demonstrated there was a linear relationship between the choroidal blood flow and the mean ocular perfusion pressure (MOPP) within a certain range in healthy subjects with normal eyes.^10^ The mean arterial pressure (MAP) was calculated from the SBP and the DBP as

MAP = DBP + 1/3(SBP − DBP).

The MOPP was calculated as

MOPP = 2/3MAP − IOP^41^

### Statistical Analyses

We evaluated the diurnal variations of parameters including the MBR using a linear mixed model to incorporate possible correlations between repeated measured values of the parameters for each eye over time and between those from the 2 eyes within a subject. This means that the statistics, linear mixed model, did not compare the values of parameters for each eye separately, and was able to evaluate the value of 1 eye to be interacted by the fellow eye over time and use the 2 eyes within a subject.

Specifically, we assumed the following model 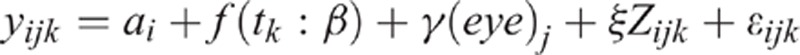


*i* (subject) = 1, ….,13, *j* (eye) = 1,2, *k* (time) = 6, 9, 12, 15, 18, 21, 24 (h)

where *y*_*ijk*_ is the MBR value for side *j* of eye (1 = right, 2 = left) at time *k* on subject *i*. *a*_*i*_ is a subject-specific random effect. The function *f*(*t*:*β*), which represents a fixed effect of time on the MBR on the ONH, was specified as a polynomial function: *f*(*t*:*β*) = *β*_*0*_* +β*_*1*_*t + β*_*2*_*t*^*2*^ +…+*β*_*p*_*t*^*p*^. The parameters *γ* and *ξ* represent fixed effects of the side of eye and a particular covariate *Z*, respectively. The order of polynomials in *f*(*t*:*β*) was selected based on the Akaike Information Criteria (AIC). For the residual term *ε*_*ijk*_ of the MBR value, we assumed a compound symmetry correlation structure within each eye.

We calculated the coefficient, 95% confidence interval, and the *P* value for the fixed effects. The level for statistical significance was 0.05. The statistical analyses were performed with SAS9.3 MIXED procedure (SAS Inc, Cary).

## RESULTS

The 13 participants had all of the examinations at the proper time without missing any.

### Diurnal Variations in MBR of Optic Nerve Head and Choroidal

**FIGURE 2:**
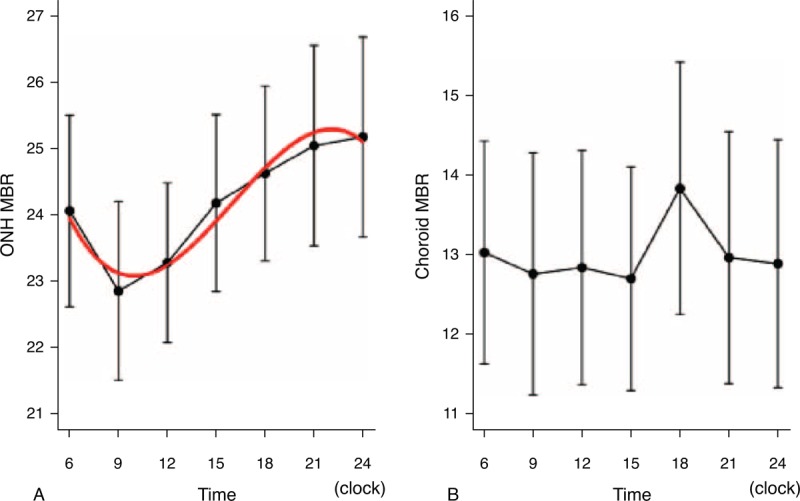
The ONH blood flow in healthy subjects had significant variations with a relative nadir MBR at 9:00 hours and progressive increase during the day to a relative peak at 24:00 hours (*P* < 0.001) (A). The best fit curve was a cubic polynomial curve (red line). The choroidal blood flow had significant diurnal fluctuations with a relative trough at 15:00 and peak at 18:00 hours (*P* = 0.001). The phase of the choroidal blood flow is different from that of ONH blood flow (B). MBR = mean blur rate, ONH =  optic nerve head.

Representative images of the diurnal variations of the MBR over the ONH and choroid obtained with the LSFG-NAVI from 1 eye are shown in Figure 1. We found that the MBR on the ONH in healthy subjects varied considerably with a relative nadir at 9:00 hours followed by a progressive increase during the day to a relative peak at 24:00 hours (*P* < 0.001). The shape of this curve can be best described by a cubic polynomial equation using linear mixed model (Table 4, Figure 2A). Although the MBR in the vessels and tissue areas on the ONH had a significant diurnal fluctuation (*P* *<* 0.001, *P* < 0.001, respectively) with same tendency as the MBR in the overall area of the ONH, the MBR in the overall area on the ONH was most significant. The MBR of the choroid also had a significant diurnal fluctuation with a relative trough at 15:00 hours and a peak at 18:00 hours (*P* = 0.001)(Figure 2B). The phase of MBR on choroid was different from that on the ONH.

**TABLE 4:**
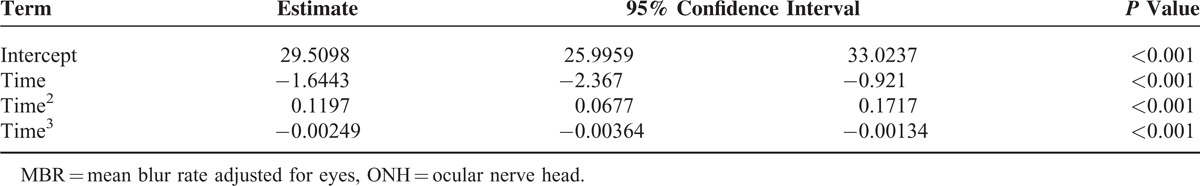
Polynomial Regression Analysis of the Relation Between ONH MBR and Time

### Diurnal Variations in Ocular and Systemic Factors, and Correlation of ONH or Choroidal MBR with Other Factors

**FIGURE 3:**
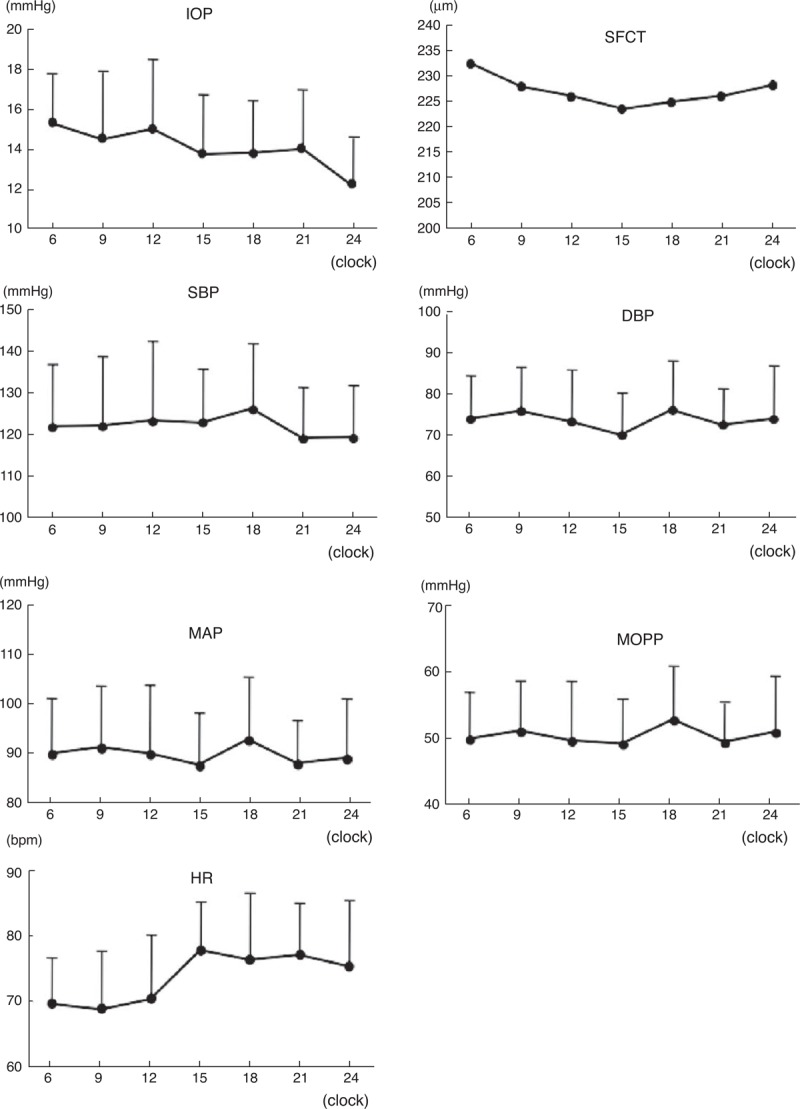
Diurnal variations in the intraocular pressure (IOP), subfoveal choroidal thickness (SFCT), systolic blood pressure (SBP), diastolic blood pressure (DBP), mean arterial pressure (MAP), mean ocular perfusion pressure (MOPP), and heat rate (HR) measured every 3 hours from 6:00 to 24:00 hours. The SBP, MAP, and MOPP had a peak at 18:00 hours, which was the same as that of the choroidal blood flow. The HR increased in the evening and the IOP was high in the morning and low at night.

The changes in the values of the ocular and systemic factors are shown in Figure 3. The IOP had a significant diurnal variation with a relative peak at 6:00 hours with a progressive decrease in the IOP during the day to a relative nadir at 12:00 hours (*P* < 0.001). The phase was different from that of the ONH (*P* = 0.539) and the choroidal MBR (*P* = 0.286).

The subfoveal choroidal thickness had a significant diurnal variation with a thinning during the day and thickening during the night (*P* < 0.001). The phase was different from that of the ONH (*P* *=* 0.702) and choroidal MBR (*P* = 0. 051). In addition, the SBP, DBP, and HR had significant diurnal variations (*P* = 0.005, *P* = 0.001, *P* < 0.001, respectively).

The trend of the systemic factors was different from that of the MBR on the ONH (Table 5). However, the phases of the SBP, MAP, and MOPP coincided with that of the MBR of the choroid with a relative peak at 18:00 hours. The SBP had the highest positive correlation with the fluctuations of the MBR of the choroid (*P* = 0.002), and both the MAP and the MOPP were positively correlated with the MBR of the choroid (*P* = 0.023, *P* = 0.047; Table 6).

**TABLE 5:**
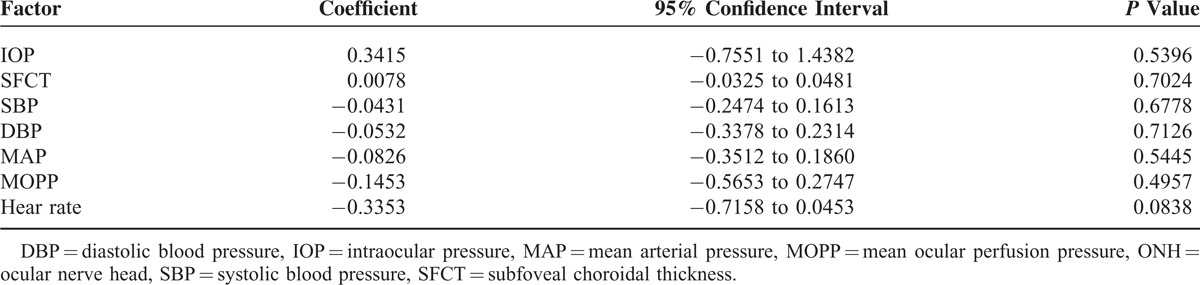
Correlation of ONH MBR With Other Factors

**TABLE 6:**
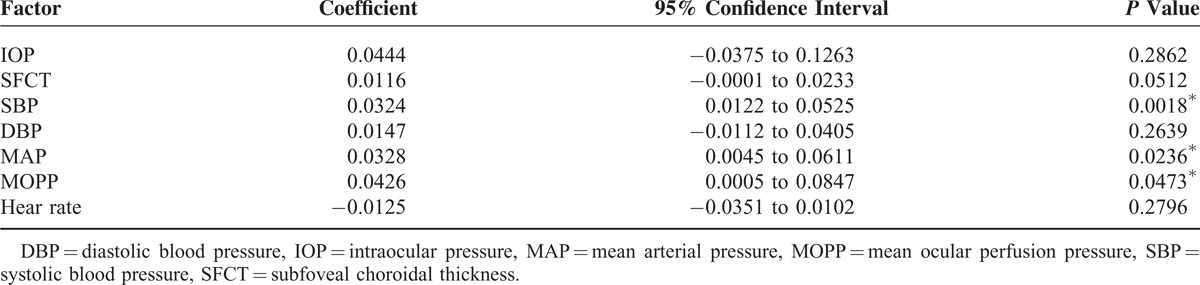
Correlation of Choroid MBR With Other Factors

## DISCUSSION

The diurnal variation of the MBR, expressing relative blood flow, on the ONH varied significantly with a trough at 9:00 hours and a peak at 24:00 hours, and the best fit curve of the diurnal variation was described by a cubic polynomial equation. In addition, the diurnal variation of MBR of the choroid also had significant diurnal variations with a trough at 15:00 hours and a peak at 18:00 hours. Earlier studies measured either the ONH or the choroidal blood flow but not both simultaneously as we did.^21,22,42,43^

The ocular blood flow has been studied with the measurements made by a variety of techniques, including laser Doppler flowmetry. However, it is somewhat difficult to follow the blood flow of the same sites at different times. On the other hand, LSFG-NAVI can measure the same areas once the landmarks are registered in the instrument (Figure 1). To the best of our best knowledge, this is the first study that measured the diurnal variations of both the ONH and choroidal blood flow simultaneously using LSFG. The results showed significant diurnal variations in the ONH and choroid blood flow in healthy subjects.

Interestingly, our analyses showed that the diurnal variations of the MBR on the ONH can be best fit by a cubic polynomial equation using a linear mixed model. Pemp et al used laser Doppler flowgraphy, and reported that larger fluctuations of the ONH blood flow were observed in patients with glaucoma than in healthy controls. However, no significant differences were found between the 2 groups.^21^ Okuno et al measured the squared blur rate, which is equal to one-half of the MBR,^34^ 3 times/day using LSFG and found significant diurnal changes in the ONH blood flow in patients with normal tension glaucoma, but no changes in the normal control group.^41^ We measured the MBR 7 different times/day from 6:00 to 24:00 hours, and the results showed that there was diurnal fluctuations which differed from those studies. The failure to detect significant changes by the earlier studies was probably because of either low sensitivity of the methods used to measure blood flow ^21^ or insufficient statistical power of the collected data.^41^

The IOP had a significant diurnal variation with a relative peak at 6:00 hours and a relative nadir at 24:00 hours which is consistent with previous reports. In addition, the SBP, DBP, HR, and choroidal thickness had significant diurnal variations. The diurnal changes of the ONH MBR were not correlated with any other factors having diurnal changes. These findings indicate that the ONH blood flow is autoregulated. It has been reported that the ONH blood flow is strongly autoregulated against fluctuations of the IOP and systemic blood pressure.^9^ In healthy subjects, it has been shown that a decrease in the MOPP did not lead to a parallel decrease in the OHN blood flow.^44,45^ In earlier studies, the ONH blood flow had some degree of autoregulation when the IOP was experimentally increased, and the ONH blood flow did not decrease significantly until the MOPP values were 30% below the baseline.^46^ In another study, a rapid increase of the IOP from 20 to either 40 or 50 mmHg induced no significant changes in the ONH blood flow in rabbits.^47^ This can be explained by the presence of autoregulation of the vascular beds at ONH, that is, the ONH blood flow was not affected by a change of the IOP and circulatory factors. Our results are consistent with those reports.

It has been reported that the superficial layers of the ONH are supplied by the central retinal artery while the deeper prelaminar regions by the posterior ciliary arteries which branch off the circle of Zinn-Haller.^46,48^ The vascular supply of the ONH is complex and the deeper vascular layers may well react differently. The wavelength used in the laser Doppler flowmeter is 811 nm, which is shorter than the 830 nm in the LSFG, and it measures the blood flow predominantly in the superficial layers of the ONH.^49^ The vasculature in the ONH is not under neural control, and, as such, it is dominated by myogenic and metabolic mechanisms when the MOPP is changed.^42^ Thus, the perfusion pressure affects the blood flow by a complex interplay of myogenic and metabolic factors.^42^ Wang et al reported that the high correlation between the blood flow in the posterior ONH but not the anterior ONH measured by LSFG and the microsphere method.^50^ These findings provide evidence that the LSFG technique is capable of assaying the blood flow in a critical region of the ONH. This region is served primarily by the short posterior ciliary circulation.^44^

Autoregulation is defined as the ability of a vascular bed to keep blood flow constant when the perfusion pressure is changed. When interpreting our results, the short posterior ciliary circulation and the myogenic and metabolic mechanisms should be correlated with the autoregulation of the ONH. This should account for the diurnal variation of the ONH blood flow.

We evaluated the MBR of the choroid at the macular area as well as the ONH. The MBR calculations we performed of the square of the macula can be considered to be mainly the choroidal blood flow, because there are no large retinal vessels found near the fovea. In addition, it has been reported that after induction of a branch retinal artery occlusion in monkey eyes, there is little change in the postinduction panoramic map when compared to the vascular patterns prior to the occlusion.^51^

We found that the choroidal blood flow in healthy subjects had a significant diurnal fluctuation with a relative trough at 15:00 hours and peak at 18:00 hours. The pattern of the diurnal fluctuations in the choroidal blood flow was totally different from that of the ONH and the IOP, but interestingly, quite similar to that of the SBP, MAP, and MOPP. The capillaries in the choroid are relatively large and fenestrated. The choroidal neurons receive parasympathetic and sympathetic innervations, and they are assumed to play a role in choroidal blood flow regulation.^4^ Our results showed significant differences between the regulatory behavior of the ONH and choroidal blood vessels when the circulatory factors were changed, which should be because of the differences between the vasculature of the ONH and the choroid. There have been reports that stated that the choroidal vessels were poorly autoregulated, and changes in the perfusion pressure directly affect the blood flow.^10,52,53^ Our results are consistent with those reports.

The subfoveal choroidal thickness increased during the night and decreased during the day, and this pattern is consistent with previous reports. The choroid is supplied by the posterior ciliary arteries, which is a branch of the ophthalmic artery, and it accounts for 85% of the total ocular blood flow to the eye.^54,55^ Usui et al reported that the subfoveal choroidal thickness was correlated with the SBP,^20^ and the choroidal blood flow was correlated with the SBP. However, Sogawa et al reported that there were no significant correlations between the subfoveal choroidal thickness and the total and subfoveal choroidal blood flows in healthy young subjects as measured by laser Doppler flowmetry.^56^ We also found that the choroidal blood flow was not significantly correlated with the subfoveal choroidal thickness.

This study has some limitations. Parameters were determined every 3 hours from 6:00 to 24:00 hours. Our study had not so many subjects and only exploratory data analysis. Nevertheless, strong significant diurnal variations were found in the ONH and choroidal blood flow. Further studies with confirmatory analysis on a large number of healthy subjects will be necessary for clarification.

In conclusion, we found significant diurnal fluctuations in the ONH and choroidal blood flow in healthy subjects by LSFG. The ONH MBR had its own diurnal variation, which was best described as a cubic polynomial equation, and it was not correlated with IOP, choroidal thickness, and systemic circulatory factors. On the other hand, the diurnal fluctuation of the choroidal blood flow was correlated with circulatory factors. Our data suggested strong complex regulation of the ONH blood flow and poor autoregulation of the choroidal blood flow. We believe that our results need to be considered when interpreting blood flow data in eyes with ocular diseases, such as those with glaucoma and AMD.

## References

[1] Chiba N Omodaka K Yokoyama Y Association between optic nerve blood flow and objective examinations in glaucoma patients with generalized enlargement disc type. *Clin Ophthalmol* 2011; 5:1549–1556. 22125400 10.2147/OPTH.S22097 PMC3218163

[2] Hayreh SS The 1994 Von Sallman Lecture. The optic nerve head circulation in health and disease. *Exp Eye Res* 1995; 61:259–272. 7556490 10.1016/s0014-4835(05)80121-6

[3] Yokoyama Y Aizawa N Chiba N Significant correlations between optic nerve head microcirculation and visual field defects and nerve fiber layer loss in glaucoma patients with myopic glaucomatous disk. *Clin Ophthalmol* 2011; 5:1721–1727. 22205831 10.2147/OPTH.S23204 PMC3245193

[4] Nickla DL Wallman J The multifunctional choroid. *Prog Retin Eye Res* 2010; 29:144–168. 20044062 10.1016/j.preteyeres.2009.12.002 PMC2913695

[5] Langham ME Kramer T Decreased choroidal blood flow associated with retinitis pigmentosa. *Eye (Lond)* 1990; 4:374–381. 2379647 10.1038/eye.1990.50

[6] Falsini B Anselmi GM Marangoni D Subfoveal choroidal blood flow and central retinal function in retinitis pigmentosa. *Invest Ophthalmol Vis Sci* 2011; 52:1064–1069. 20861481 10.1167/iovs.10-5964

[7] Fontana L Poinoosawmy D Bunce CV Pulsatile ocular blood flow investigation in asymmetric normal tension glaucoma and normal subjects. *Br J Ophthalmol* 1998; 82:731–736. 9924361 10.1136/bjo.82.7.731 PMC1722652

[8] Ravalico G Toffoli G Pastori G Age-related ocular blood flow changes. *Invest Ophthalmol Vis Sci* 1996; 37:2645–2650. 8977478

[9] Okuno T Oku H Sugiyama T Evidence that nitric oxide is involved in autoregulation in optic nerve head of rabbits. *Invest Ophthalmol Vis Sci* 2002; 43:784–789. 11867599

[10] Riva CE Titze P Hero M Effect of acute decreases of perfusion pressure on choroidal blood flow in humans. *Invest Ophthalmol Vis Sci* 1997; 38:1752–1760. 9286263

[11] Kitazawa Y Horie T Diurnal variation of intraocular pressure in primary open-angle glaucoma. *Am J Ophthalmol* 1975; 79:557–566. 1168023 10.1016/0002-9394(75)90792-8

[12] Liu JHK Kripke DF Hoffman RE Nocturnal elevation of intraocular pressure in young adults. *Invest Ophthalmol Vis Sci* 1998; 39:2707–2712. 9856781

[13] Wilson LB Quinn GE Ying G The relation of axial length and intraocular pressure fluctuations in human eyes. *Invest Ophthalmol Vis Sci* 2006; 47:1778–1784. 16638981 10.1167/iovs.05-0869

[14] Read SA Collins MJ Iskander DR Diurnal variation of axial length, intraocular pressure, and anterior eye biometrics. *Invest Ophthalmol Vis Sci* 2008; 49:2911–2918. 18362106 10.1167/iovs.08-1833

[15] Kiely PM Carney LG Smith G Diurnal variations of corneal topography and thickness. *Am J Optom Physiol Opt* 1982; 59:976–982. 6891565 10.1097/00006324-198212000-00007

[16] Harper CL Boulton ME Bennett D Diurnal variations in human corneal thickness. *Br J Ophthalmol* 1996; 80:1068–1072. 9059272 10.1136/bjo.80.12.1068 PMC505705

[17] Read SA Collins MJ Diurnal variation of corneal shape and thickness. *Optom Vis Sci* 2009; 86:170–180. 19182699 10.1097/OPX.0b013e3181981b7e

[18] Read SA Collins MJ Carney LG The diurnal variation of corneal topography and aberrations. *Cornea* 2005; 24:678–687. 16015086 10.1097/01.ico.0000154385.43732.6e

[19] Mapstone R Clark CV Diurnal variation in the dimensions of the anterior chamber. *Arch Ophthalmol* 1985; 103:1485–1486. 4051851 10.1001/archopht.1985.01050100061019

[20] Usui S Ikuno Y Akiba M Circadian changes in subfoveal choroidal thickness and the relationship with circulatory factors in healthy subjects. *Invest Ophthalmol Vis Sci* 2012; 53:2300–2307. 22427554 10.1167/iovs.11-8383

[21] Pemp B Georgopoulos M Vass C Diurnal fluctuation of ocular blood flow parameters in patients with primary open-angle glaucoma and healthy subjects. *Br J Ophthalmol* 2009; 93:486–491. 19029154 10.1136/bjo.2008.148676

[22] Sehi M Flanagan JG Zeng L Anterior optic nerve capillary blood flow response to diurnal variation of mean ocular perfusion pressure in early untreated primary open-angle glaucoma. *Invest Ophthalmol Vis Sci* 2005; 46:4581–4587. 16303952 10.1167/iovs.05-0209

[23] Riva CE Feke GT Ben-Sira I Fluorescein dye-dilution technique and retinal circulation. *Am J Physiol* 1978; 234:315–322. 10.1152/ajpheart.1978.234.3.H315 343603

[24] Hales JR Radioactive microsphere measurement of cardiac output and regional tissue blood flow in the sheep. *Pflugers Arch* 1973; 344:119–132. 4797947 10.1007/BF00586546

[25] Pasztor E Symon L Dorsch NW The hydrogen clearance method in assessment of blood flow in cortex, white matter and deep nuclei of baboons. *Stroke* 1973; 4:556–567. 4198833 10.1161/01.str.4.4.556

[26] Riva C Ross B Benedek GB Laser Doppler measurements of blood flow in capillary tubes and retinal arteries. *Invest Ophthalmol* 1972; 11:936–944. 4634958

[27] Feke GT Tagawa H Deupree DM Blood flow in the normal human retina. *Invest Ophthalmol Vis Sci* 1989; 30:58–65. 2643588

[28] Tamaki Y Araie M Kawamoto E Noncontact, two-dimensional measurement of retinal microcirculation using laser speckle phenomenon. *Invest Ophthalmol Vis Sci* 1994; 35:3825–3834. 7928179

[29] Tamaki Y Araie M Tomita K Effect of topical timolol on tissue circulation in optic nerve head. *Jpn J Ophthalmol* 1997; 41:297–304. 9363557 10.1016/s0021-5155(97)00054-3

[30] Nagahara M Tamaki Y Tomidokoro A In vivo measurement of blood velocity in human major retinal vessels using the laser speckle method. *Invest Ophthalmol Vis Sci* 2011; 52:87–92. 20702824 10.1167/iovs.09-4422

[31] Sugiyama T Araie M Riva CE Use of laser speckle flowgraphy in ocular blood flow research. *Acta Ophthalmol* 2010; 88:723–729. 19725814 10.1111/j.1755-3768.2009.01586.x

[32] Houle RE Grant WM Alcohol, vasopressin, and intraocular pressure. *Invest Ophthalmol* 1967; 6:145–154. 6022594

[33] Avisar R Avisar E Weinberger D Effect of coffee consumption on intraocular pressure. *Ann Pharmacother* 2002; 36:992–995. 12022898 10.1345/aph.1A279

[34] Fujii H Visualization of retinal blood flow by laser speckle flowgraphy. *Med Biol Eng Comput* 1994; 32:302–304. 7934254 10.1007/BF02512526

[35] Sugiyama T Utsumi T Azuma I Measurement of optic nerve head circulation: comparison of laser speckle and hydrogen clearance methods. *Jpn J Ophthalmol* 1996; 40:339–343. 8988423

[36] Tamaki Y Araie M Kawamoto E Noncontact, two-dimensional measurement of tissue circulation in choroid and optic nerve head using laser speckle phenomenon. *Exp Eye Res* 1995; 60:373–384. 7789417 10.1016/s0014-4835(05)80094-6

[37] Tamaki Y Araie M Tomita K Real-time measurement of human optic nerve head and choroid circulation using the laser speckle phenomenon. *Jpn J Ophthalmol* 1997; 41:49–54. 9147189 10.1016/s0021-5155(96)00008-1

[38] Fujii H Konishi N Okamoto K A new version of real-time laser flowgraphy. *Atarashii Ganka (Eye)* 1996; 13:957–961.

[39] Konishi N Tokimoto Y Kohra K New laser speckle flowgraphy system using CCD camera. *Opt Rev* 2002; 9:163–169.

[40] Spaide RF Koizumi H Pozzoni MC Enhanced depth imaging spectral-domain optical coherence tomography. *Am J Ophthalmol* 2008; 146:496–500. 18639219 10.1016/j.ajo.2008.05.032

[41] Okuno T Sugiyama T Kojima S Diurnal variation in microcirculation of ocular fundus and visual field change in normal-tension glaucoma. *Eye (Lond)* 2004; 18:697–702. 14739923 10.1038/sj.eye.6700749

[42] Schmidl D Garhofer G Schmetterer L The complex interaction between ocular perfusion pressure and ocular blood flow—relevance for glaucoma. *Exp Eye Res* 2011; 93:141–155. 20868686 10.1016/j.exer.2010.09.002

[43] Pournaras CJ Rungger-Br̈andle E Riva CE Regulation of retinal blood flow in health and disease. *Prog Retin Eye Res* 2008; 27:284–330. 18448380 10.1016/j.preteyeres.2008.02.002

[44] Pillunat LE Anderson DR Knighton RW Autoregulation of human optic nerve head circulation in response to increased intraocular pressure. *Exp Eye Res* 1997; 64:737–744. 9245904 10.1006/exer.1996.0263

[45] Weigert G Findl O Luksch A Effects of moderate changes in intraocular pressure on ocular hemodynamics in patients with primary open-angle glaucoma and healthy controls. *Ophthalmology* 2005; 112:1337–1342. 16024084 10.1016/j.ophtha.2005.03.016

[46] Schmidl D Boltz A Kaya S Role of nitric oxide in optic nerve head blood flow regulation during isometric exercise in healthy humans. *Invest Ophthalmol Vis Sci* 2013; 54:1964–1970. 23439596 10.1167/iovs.12-11406

[47] Takayama J Tomidokoro A Ishii K Time course of the change in optic nerve head circulation after an acute increase in intraocular pressure. *Invest Ophthalmol Vis Sci* 2003; 44:3977–3985. 12939318 10.1167/iovs.03-0024

[48] Caprioli J Coleman AL Blood pressure, perfusion pressure, and glaucoma. *Am J Ophthalmol* 2010; 149:704–712. 20399924 10.1016/j.ajo.2010.01.018

[49] Petrig BL Riva CE Hayreh SS Laser Doppler flowmetry and optic nerve head blood flow. *Am J Ophthalmol* 1999; 127:413–425. 10218694 10.1016/s0002-9394(98)00437-1

[50] Wang L Cull GA Piper C Anterior and posterior optic nerve head blood flow in nonhuman primate experimental glaucoma model measured by laser speckle imaging technique and microsphere method. *Invest Ophthalmol Vis Sci* 2012; 53:8303–8309. 23169886 10.1167/iovs.12-10911 PMC3525139

[51] Alm A Bill A Ocular and optic nerve blood flow at normal and increased intraocular pressures in monkeys (Macaca irus): a study with radioactively labelled microspheres including flow determinations in brain and some other tissues. *Exp Eye Res* 1973; 15:15–29. 4630581 10.1016/0014-4835(73)90185-1

[52] Kiel JW Modulation of choroidal autoregulation in the rabbit. *Exp Eye Res* 1999; 69:413–429. 10504275 10.1006/exer.1999.0717

[53] Delaey C Van De Voorde J Regulatory mechanisms in the retinal and choroidal circulation. *Ophthalmic Res* 2000; 32:249–256. 11015035 10.1159/000055622

[54] Flugel C Tamm ER Mayer B Species differences in choroidal vasodilative innervation: evidence for specific intrinsic nitrergic and VIP-positive neurons in the human eye. *Invest Ophthalmol Vis Sci* 1994; 35:592–599. 7509326

[55] Flugel-Koch C May CA Lutjen-Drecoll E Presence of a contractile cell network in the human choroid. *Ophthalmologica* 1966; 210:296–302. 8878213 10.1159/000310728

[56] Sogawa K Nagaoka T Takahashi A Relationship between choroidal thickness and choroidal circulation in healthy young subjects. *Am J Ophthalmol* 2012; 153:1129–1132. 22310083 10.1016/j.ajo.2011.11.005

